# Association Mapping and Validation of QTLs for Flour Yield in the Soft Winter Wheat Variety Kitahonami

**DOI:** 10.1371/journal.pone.0111337

**Published:** 2014-10-31

**Authors:** Goro Ishikawa, Kazuhiro Nakamura, Hiroyuki Ito, Mika Saito, Mikako Sato, Hironobu Jinno, Yasuhiro Yoshimura, Tsutomu Nishimura, Hidekazu Maejima, Yasushi Uehara, Fuminori Kobayashi, Toshiki Nakamura

**Affiliations:** 1 NARO Tohoku Agricultural Research Center, Morioka, Iwate, Japan; 2 NARO Kyusyu Okinawa Agricultural Research Center, Chikugo, Fukuoka, Japan; 3 Kitami Agricultural Experiment Station, Hokkaido Research Organization, Tokoro-gun, Hokkaido, Japan; 4 Central Agricultural Experiment Station, Hokkaido Research Organization, Yubari-gun, Hokkaido, Japan; 5 Kamikawa Agricultural Experiment Station, Hokkaido Research Organization, Kamikawa-gun, Hokkaido, Japan; 6 Nagano Agricultural Experiment Station, Suzaka, Nagano, Japan; 7 National Institute of Agrobiological Sciences, Kannondai, Tsukuba, Japan; Huazhong University of Science and Technology, China

## Abstract

The winter wheat variety Kitahonami shows a superior flour yield in comparison to other Japanese soft wheat varieties. To map the quantitative trait loci (QTL) associated with this trait, association mapping was performed using a panel of lines from Kitahonami’s pedigree, along with leading Japanese varieties and advanced breeding lines. Using a mixed linear model corrected for kernel types and familial relatedness, 62 marker-trait associations for flour yield were identified and classified into 21 QTLs. In eighteen of these, Kitahonami alleles showed positive effects. Pedigree analysis demonstrated that a continuous pyramiding of QTLs had occurred throughout the breeding history of Kitahonami. Linkage analyses using three sets of doubled haploid populations from crosses in which Kitahonami was used as a parent were performed, leading to the validation of five of the eight QTLs tested. Among these, QTLs on chromosomes 3B and 7A showed highly significant and consistent effects across the three populations. This study shows that pedigree-based association mapping using breeding materials can be a useful method for QTL identification at the early stages of breeding programs.

## Introduction

Flour yield, or the percentage of flour from a given quantity of grain, is of great importance to flour milling companies. Flour yield can be increased by the enhancement of techniques in the milling process, or through the development of varieties with higher flour yields. In 2006, the soft winter wheat variety Kitahonami was released in the Hokkaido prefecture of Japan [1]. This variety, which has become a leading variety in Hokkaido, shows the highest flour yield among Japanese soft wheat varieties. Therefore, Kitahonami is now being used as a source of the high flour-yield trait in multiple Japanese wheat breeding programs. Mapping of quantitative trait loci (QTL) associated with this trait and identification of linked markers would accelerate the introgression of the high flour-yield phenotype into other varieties.

However, flour yield is a complex trait that appears to be strongly influenced by genetic background. QTL studies using bi-parental populations have been conducted within hard varieties or within populations of interclass hybridizations between hard and soft varieties. These studies have indicated that QTLs for flour yield are located on 16 out of 21 chromosomes: 1B, 1D, 2A, 2B, 3A, 3B, 4A, 4B, 4D, 5A, 5B, 5D, 6B, 6D, 7A and 7D [2]–[6]. Interclass hybridization between soft and hard wheat demonstrated that the hardness locus *Pinb* on 5D chromosome had a strong influence on flour yield [3]. In soft wheat types, only a limited number of studies identifying QTLs associated with flour yield have been reported to date. Using an association mapping approach with 95 soft wheat varieties, Breseghello and Sorrells [7] detected weak QTLs associated with flour yield and break flour yield on 2D and 5B. A study of a bi-parental population derived from two soft wheat cultivars identified QTLs for flour yield on 1B, 2A, 2B, 2D and 3B [8]. Carter et al. [9] found that a large number of QTLs for milling quality and starch functionality were located on 3B and 4D, including QTLs for flour yield. Although the QTLs described in these studies were detected with high confidence, few were consistent between studies, suggesting that they are unlikely to coincide with the high flour yield trait from Kitahonami.

Because developing mapping populations and performing mapping studies are time consuming processes, breeders often have already introgressed target QTL into breeding lines using traditional selection methods before markers are available, especially for highly desirable traits. Thus, the most effective stage for using marker-assisted selection (MAS) to introduce a new trait into breeding programs is often missed. One solution to this could be to take advantage of populations developed within a breeding program to identify QTLs. Jannink et al. [10] proposed an approach applying family-based methods that are generally used within human and animal populations. Family-based QTL mapping for resistance to Fusarium head blight has been reported [11], and Malosetti et al. [12] used pedigree-data in association mapping of resistance to *Phytophthora infestans* in potato. Such association mapping techniques might be useful in rapid marker development for MAS.

The objective of this study was to dissect the genetic factors contributing to the high flour-yield trait of Kitahonami by an association mapping approach. After identification of QTLs related to flour yield, the pedigree record of Kitahonami was used to trace the origin of QTLs which had been inadvertently accumulated through selection for high flour yield. To confirm the utility of this approach, QTLs identified by association mapping were validated using our own bi-parental populations.

## Materials and Methods

Plant materials: One hundred eighty-five accessions were used in this study (Table S1 in File S1). Of these, 65 accessions were winter wheat varieties related to Kitahonami and lines from the pedigree of Kitahonami (Fig. S1), along with advanced breeding materials and varieties developed at Kitami Agricultural Experimental Station (KAES), NARO Tohoku Agricultural Research Center (TARC) and Nagano Agricultural Experimental Station (NAES). These lines, which made up the association panel, were subjected to intensive phenotyping and were used in an association analysis. The remaining 120 accessions, which were included in a diversity analysis to investigate the genetic diversity of Japanese breeding materials, consisted of leading varieties and advanced breeding lines from across the country, along with introduced varieties from other countries and experimental lines such as Chinese Spring and *T. spelta* var. *duhamelianum*.

DNA isolation and genome-wide marker analysis: Genomic DNA of each accession was extracted from 100 mg of young leaf tissue using the automated DNA isolation systems PI-50α or PI-80X (Kurabo Industries Ltd., Osaka, Japan) according to the manufacturer’s instructions. For the diversity analysis, 151 accessions were genotyped by DArT [Wheat *Pst*I (*Taq*I) v.3] (Diversity Arrays Technology Pty Ltd., http://www.diversityarrays.com/) and 164 accessions were genotyped by SNP (PrivKSU_WheatCons_9k) [13] markers. After removing data with minor allele frequencies (MAF) of less than 0.01, genotyping data from 2,933 DArT and 6,042 SNP markers were forwarded for diversity analysis. In addition to DArT and SNP genotyping, materials in the association panel were also genotyped using SSR markers (GrainGenes 2.0, http://wheat.pw.usda.gov/GG2/index.shtml/) and established diagnostic markers, such as *Pina-D1*, *Pinb-D1*, *Wx-A1*, *Wx-B1*, *Ppo-A1*, *Ppo-D1*, *Psy-A1* and *Psy-B1* (reviewed in Liu et al. [14]). Genotyping data from SSR markers was recorded in the bi-allelic state: each fragment derived from a SSR marker was recorded as presence (1) or absence (0). All genotyping data from the association panel was merged. Data with MAF of less than 0.1 and redundancies among markers were removed. After these processes, genotyping data from 3,815 selected markers was used for the association analysis. Distribution of these markers across the wheat genome is shown in Table S2 in File S1.

Field experiments: Accessions in the association panel were field-grown in three locations [Kitami (Hokkaido island, 43.7°N, 143.7°E), Morioka (Northern Honsyu island, 39.7°N, 141.1°E) and Nagano (Central of Honsyu island, 36.7°N, 138.3°E)], during the three successive cropping seasons from 2008/2009 to 2010/2011. The plot size was 3.0 m×0.7 m, and each plot consisted of 40–50 plants separated from one another by 10–15 cm. Two replications were conducted in each season except 2008/2009.

Trait analyses: Grain samples were tempered to 14.5% moisture and 100 g of each sample was milled on a Quadrumat Junior mill (Brabender Co., Hackensack, NJ). The mill was preheated to prevent expansion of the rolls during operation. Ground grain samples were sifted with an 8XX silk reel sieve and a 94-mesh (180 µm) screen. Flour yield (FlYd) was expressed as the percentage of total flour weight to initial sample weight. Measurements were also taken for the following 14 traits: flour efficiency (FlEf), median size of flour particles (x50), specific surface area of flour particles (Sv), flour protein content (FPC), flour ash content (Fash), flour color L* (FlL), flour color a* (Fla), flour color b* (Flb), grain protein content (GPC), grain ash content (Gash), test weight (TestW), 1000-kernel weight (TKW), heading date (HD) and maturity date (MD). Detailed explanations for each trait are described in Table S3 in File S1.

Statistical analysis: Principal component analysis (PCA) and cladogram construction were performed with TASSEL 3.0 [15]. Analyses of DArT and SNP data were conducted separately. A correlation-based PCA was performed, and missing data was imputed with following settings: use manhatten distance, use unweighted average, 3 numbers of neighbors, and 0.80 minimum frequency of row data. Statistical analysis of traits was performed with JMP 9 (SAS Institute, Raleigh, NC). The mean value of two replications was used as the environmental value for each accession in the 2009/2010 and 2010/2011 cropping seasons. For two-dimensional analysis of variance, the fit model function was used with standard least squares method. Random effects of the genotypes and environments were applied to estimate the variance components. Heritability in the broad sense was estimated from the results of the variance analysis according to the formula used by Burton and DeVane [16]. Associations between markers and traits were calculated with TASSEL 3.0 [15] using the mixed linear model. The kinship matrix calculated by TASSEL was used for considering familial relatedness of accessions. Since a different distribution pattern was observed between soft and hard kernel types (see Results' section), the effect of kernel type was considered as an additional term of fixed effect in the model: 0 and 1 values were rendered to soft and hard kernel type, respectively. To take into account multiple comparisons, significance was tested using a 0.5 false discovery rate implemented in the q value software [17].

QTL validation: QTLs obtained as described above were validated using three doubled haploid (DH) populations, which were developed from F_1_ plants from crosses between Kitahonami and three other varieties, namely Kinuhime, Tohoku224 and Shunyou. At least 151 lines from each population were field-grown without replication during the 2010/2011 season and subjected to validation. FlYd values for these lines were obtained as described above. To reduce genotyping costs, markers showing significant association with this trait in the association mapping analysis were converted from array-based SNP markers into PCR-based markers. To do this, probe sequences of the SNPs of interest were identified; since most of these sequences have been mapped, the chromosome number to which they have been assigned is known. These sequences were used as queries in BLASTN searches (E-value<e-40) against the wheat survey sequences (IWGSC, http://www.wheatgenome.org/) [18]. Generally, this allowed the identification of three highly homologous contigs from the relevant A, B and D homoeologous chromosomes, one of which showed 100% match to the probe sequence and originated from the same chromosome to which the probe sequence had been mapped. Contigs were aligned and regions that were polymorphic among the three genomes were used to design genome-specific primers (GSPs) upstream and downstream from the SNP of interest, with the SNP location set at approximately one third of the interval between primers. Two additional allele-specific primers (ASPs) were designing, with the 3′ base of these primers being concurrent with the SNP. To increase allele specificity, an artificial mismatch was integrated at the third nucleotide from the 3′ end of the ASPs, as described by Liu et al. [19]. Each PCR reaction included both GSPs and one ASP, and two reactions, each containing a different ASP, were used for genotyping. For PCR analysis, each 25-µL PCR mixture included 50–100 ng of DNA, 1.5 mM MgCl_2_, 0.2 mM dNTP (each), 1×Ex Taq buffer, and 0.5 U of TaKaRa Ex Taq (Takara, Osaka, Japan). The concentrations of GSPs and ASPs in PCR mixtures are provided in the Table S4 in File S1. The PCR cycle consisted of an initial 5 min denaturation at 95°C, followed by 32 cycles of 95°C for 30 s, 55 −62°C for 30 s, and 72°C for 1 min, followed by a final extension at 72°C for 7 min. PCR products were separated by electrophoresis on a QIAxcel system (Qiagen, Hilden, Germany) using a QIAxcel DNA screening kit. Differences between allele mean values were tested by a T-test function implemented in JMP 9 for each combination of QTL and population. As well, the effects of the eight selected markers on FlYd were estimated using a multiple regression model. The fit model function of JMP 9 was used with the standard least squares method. Regression models were constructed based on the three combined DH populations (DH_total) or on each population separately (DH_each). The models were used to predict FlYd values of the individual DH lines. Correlation coefficients between predicted and actual values were determined.

## Results

### Population structure and familial relationships

**Figure 1:**
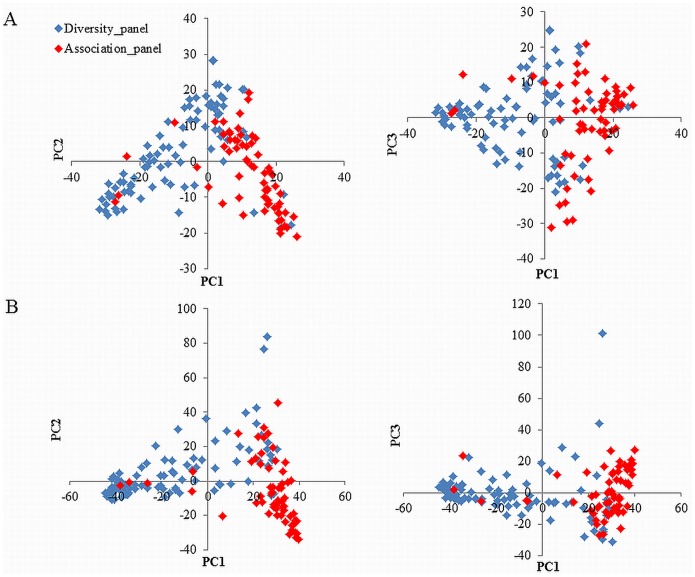
Scatter diagrams of principal component (PC) 1, 2 and 3 values calculated by the PCA function of TASSEL 3.0 using 2,933 DArT (A) and 6,042 SNP (B) markers.

Array-based marker analyses allowed the identification of 2,933 polymorphic DArT (Dataset S1 in File S2) and 6,042 polymorphic SNP markers (Dataset S2 in File S2) using 151 and 164 accessions, respectively. To obtain an overview of the genetic diversity of the accessions, a PCA was performed with each marker type. With the DArT markers, principal component (PC) 1, PC2 and PC3 explained 9.0, 4.6 and 3.9% of the total variation, while the first three PCs from the SNP markers explained 15.0, 5.2 and 3.5% of the variation. Scatter plots of PC1 and either PC2 or PC3 showed similar distribution patterns for both marker types (Fig. 1). These plots indicated that the accessions were distributed continuously and did not form any clear clusters. For the association panel, most accessions showed high PC1 values, but no clear tendencies were observed with PC2 and PC3 values. Based on the source of the accessions, PC1 represents the axis of earliness or growth habit (data not shown). In the scatter plots of SNP markers, two accessions, U24 (Acc. no. 114) and Gabo (124), showed outlier values in PC2 and PC3. This indicates that the SNP markers used in this study have more power to distinguish lines than the DArT markers.

**Figure 2:**
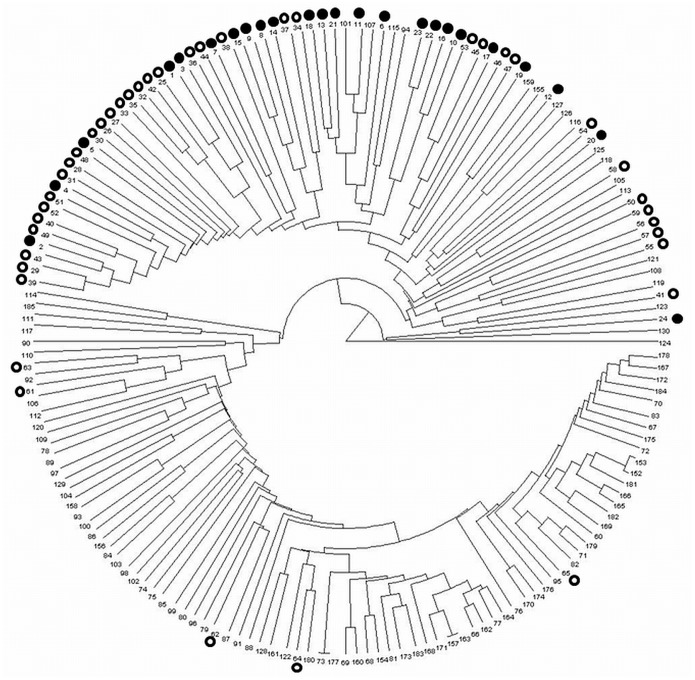
UPGMA dendrogram showing the pattern of genetic diversity among the 164 accessions based on the analysis of 6,042 SNP markers. Open and black circles indicate accessions found in the Association panel. Black circle means accessions in Kitahonami's pedigree. Numbers outside of the dendrogram correspond to accession numbers in Table S1 in File S1.

To investigate relationships among accessions, a cladogram was generated based on a distance matrix. Little difference was observed between the marker types, therefore only the cladogram generated from the SNP genotyping was employed here. As shown in Fig. 2, accessions were classified into two main clusters, and most accessions in the association panel into the same cluster. Particularly, clusters within the first four nodes from Kitahonami (Acc. no. 1) displayed relatively short distances and contained 35.4% (23/65) of accessions in the association panel. This indicates that familial relationship should be taken into account for association mapping.

### Flour yield and relationship with other traits

**Figure 3:**
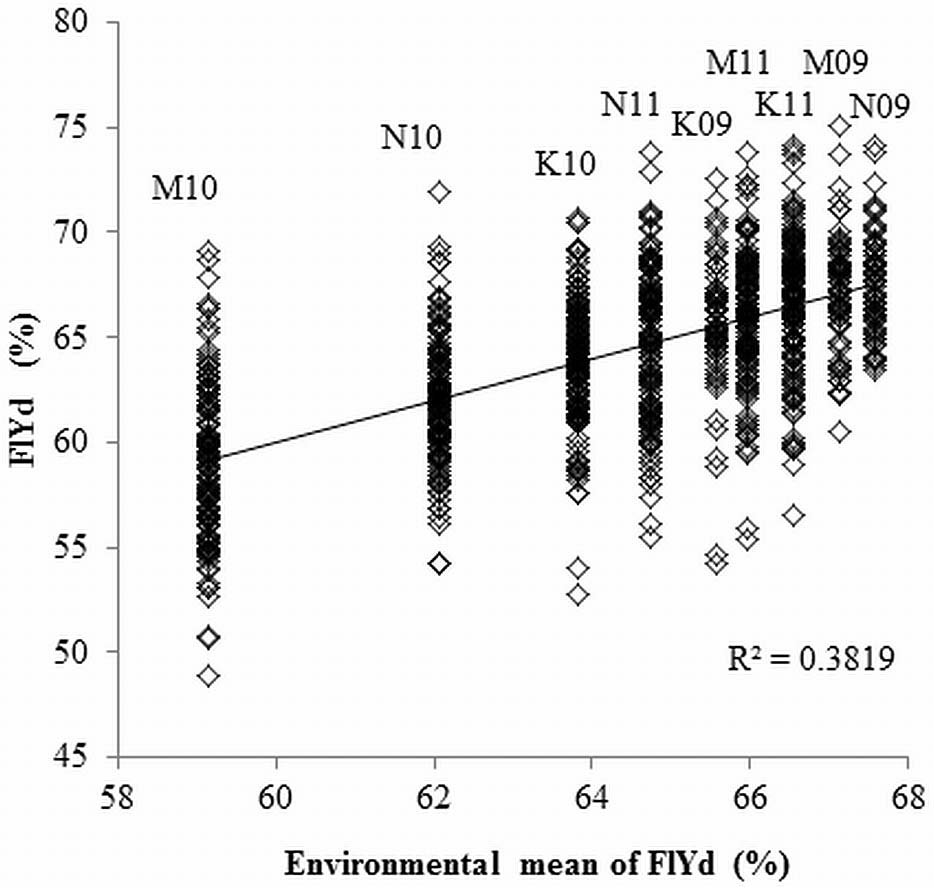
Leverage plots of flour yield (FlYd) values. Abbreviations for environments consist of first letter of location and harvest year. K: Kitami, M: Morioka, N: Nagano. For example, K09 means samples of 2008/2009 cropping season at Kitami.

The FlYd of lines in each environment and their mean values over the nine environments are provided in Dataset S3 in File S2. The analysis of variance showed significant genetic and environmental variation in FlYd compared to residual errors. Mean squares of accessions and environments were 61.76 (F = 19.65***) and 475.57 (F = 151.26***), respectively. The heritability of FlYd was 38.5%, which was relatively low compared to other traits investigated (Table S5 in File S1). This indicates that environmental factors have strong influence on FlYd. Leverage plots of environment also indicated a significant environmental effect on FlYd (Fig. 3). Samples harvested in 2010 showed a lower mean value for FlYd in all locations, and the lowest mean value was observed in the samples from Morioka in 2010. However, correlations across nine environments ranged from 0.419 to 0.945 (average 0.717), indicating that relative differences among accessions were consistent over the environments (Table 1). Therefore, we considered the mean value from the nine environments as the genotypic value of each accession.

**Table 1:** Correlation coefficients among environments for flour yield (FlYd).

Harvest year	Location	2009			2010			2011			Mean
		Kitami	Morioka	Nagano	Kitami	Morioka	Nagano	Kitami	Morioka	Nagano	
2009	Kitami		0.591	0.492	0.624	0.575	0.517	0.635	0.624	0.419	0.723
	Morioka			0.630	0.800	0.828	0.769	0.817	0.849	0.562	0.893
	Nagano				0.613	0.577	0.664	0.566	0.694	0.522	0.739
2010	Kitami					0.791	0.733	0.892	0.887	0.682	0.921
	Morioka						0.669	0.773	0.829	0.693	0.888
	Nagano							0.745	0.769	0.552	0.832
2011	Kitami								0.892	0.688	0.920
	Morioka									0.688	0.945
	Nagano										0.772

**Figure 4:**
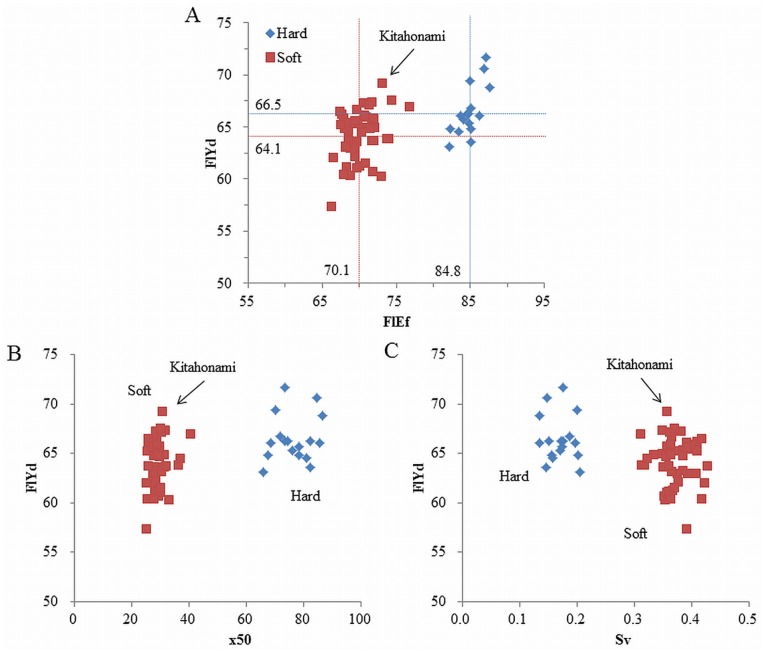
Relationships between flour yield (FlYd) and flour efficiency (FlEf) (A), FlYd and median diameter of particles (x50) (B) and FlYd and specific surface area of particles (Sv) (C). Accessions could be classified into either soft or hard kernel types based on *Pina-D1*/*Pinb-D1* genotypes. Soft accessions have *Pina-D1a* and *Pinb-D1a*, while hard have *Pina-D1b* or *Pinb-D1b*.

Relationships between FlYd and other quality traits were also investigated (TableS6 in File S1). The FlEf (r = 0.511), x50 (0.436), Sv (−0.430) and Fash (0.349) each showed a significant relationship with FlYd, while no relationship with the other traits investigated was observed. It has been reported that x50, Sv and FlEf, as well as FlYd, have strong correlations with soft and hard kernel types (reviewed in Morris [20]). Therefore, the kernel types of 65 accessions were genotyped by *Pina-D1*/*Pinb-D1* markers [21], [22]. All were classified as either soft (48) or hard (17) type. Taking kernel type into consideration, the relationships between FlYd and the other four traits were reanalyzed. This revealed two clear clusters attributable to kernel type, and no correlation with FlYd was detected in either cluster; only FlEf was correlated with kernel type (Fig. 4; TableS6 in File S1). Kitahonami showed the highest FlYd value among accessions in the soft cluster, although its value was considerably lower than the highest value observed in the hard cluster. These results clearly indicate that kernel type is an important element to consider in the association analysis.

### Association analysis for flour yield using mixed-model

**Figure 5:**
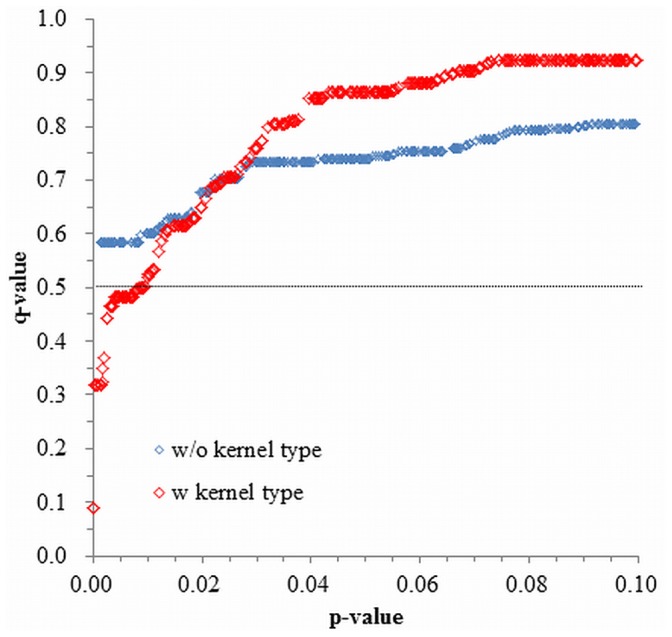
Distributions of p- and q-values and impact of kernel type correction on association mapping results for flour yield (FlYd). The distribution was calculated without (w/o) or with (w) employing kernel type as a covariant.

Genotype data obtained with the 3,815 selected markers was used for association mapping (Dataset S4 in File S2). Calculations were performed using a mixed linear model, with and without using kernel type as a covariant. To take into account multiple comparisons, a false discovery rate (q value) was adopted in determining significant marker-trait associations (MTAs). Distributions of q values with and without using kernel type as a covariant are shown in Fig. 5. When the kernel type was not used in the model, the q value was within 0.55–0.85. When kernel type was accounted for, more accurate MTAs were detected, as indicated by q values ranging from 0.09 to 0.93. Therefore, to select reliable markers, kernel type was used as a covariant and the threshold of the q value was set at 0.5. This led to the identification of a total of 62 markers (Table S7 in File S1). Based on the locations of the markers [13], [23], MTAs were classified into 21 QTLs (Table 2), although five MTA locations remained undetermined (Table S7 in File S1). Among the 21 QTLs, 18 had positive effects when the Kitahonami allele was present (Table 2). Since QTLs were classified based on two consensus genetic maps, it is possible that some QTLs overlapped: for example, 3B.3 may represent the same QTL as 3B.1 or 3B.2 (Table 2). Among the 62 MTAs, r^2^ ranged from 9.2 to 20.5% and effects on FlYd ranged from 1.51 to 2.68 (Table S7 in File S1).

**Table 2:** Flour yield (FlYd) QTLs detected by genome-wide association mapping.

QTL	No of markers	Mean of markers within QTL				Map^c^	Linkage group	(cM)
		MAF^a^	q-value	R^2^ (%)	Effect^b^			
1B.1	1	0.117	0.319	17.0	−2.572	DArT	1B	17.5
1B.2	1	0.316	0.482	11.5	1.846	DArT	1B	40.3
2B.1	2	0.359	0.482	9.4	1.755	SNP	2B	56.2
2B.2	5	0.379	0.322	14.4	2.031	SNP	2B	210.2–217.2
3B.1	4	0.335	0.437	10.9	1.897	SNP	3B	61.0–71.7
3B.2	1	0.313	0.482	9.3	2.022	SNP	3B	91.1
3B.3	1	0.270	0.482	9.3	1.614	DArT	3B	53.2
3D	1	0.346	0.482	11.0	1.708	DArT	3D	51.0
4B	1	0.238	0.482	10.3	1.851	DArT	4B	20.4
5A	1	0.234	0.443	11.3	2.180	SNP	5A	27.8
5B	1	0.156	0.482	9.7	−2.119	SNP	5B	212.5
5D.1	4	0.359	0.431	11.7	1.910	SNP	5D1cult	42.5–47.6
5D.2	4	0.188	0.482	9.5	2.172	SNP	5D3cult	8.4–10.6
6A.1	1	0.219	0.319	14.5	2.676	SNP	6A	75.5
6A.2	21	0.322	0.385	11.5	1.999	SNP	6A	114.5–117.0
6B	1	0.387	0.482	9.8	1.750	SNP	6B	59.5
7A	2	0.211	0.415	10.6	2.343	SNP	7A	58.6
7B.1	1	0.266	0.466	11.0	1.914	SNP	7B	73.3
7B.2	2	0.198	0.400	11.3	2.167	SNP	7B	115.9
7B.3	1	0.203	0.482	10.3	−2.104	SNP	7B	164.9
7D	1	0.220	0.466	11.4	2.353	DArT	7D	1.6

a Minor allele frequency.

b Increasing values indicate increasing effect of Kitahonami alleles.

c SNP and DArT locations are based on the consensus map of Cavanagh et al. [13] and Huang et al. [23], respectively.

### Pedigree analysis for flour yield QTL

**Figure 6:**
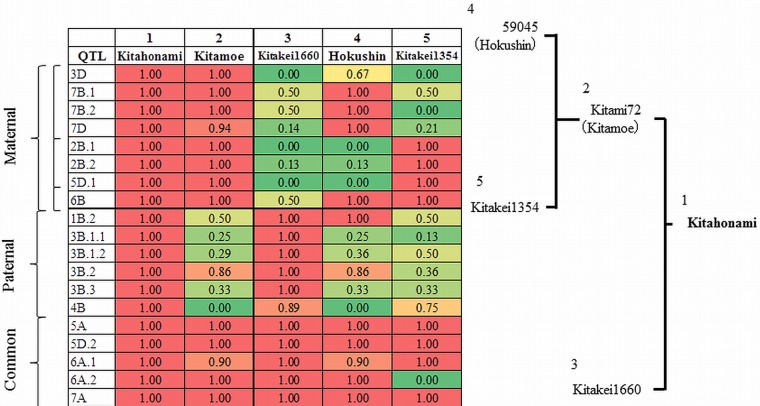
Pedigree analysis of flour yield (FlYd) QTLs. Values indicate frequencies of the same genotype as Kitahonami for each QTL.

For the 18 QTLs that showed positive effects on FlYd when Kitahonami alleles were present, linkage disequilibrium (LD) analyses of the associated loci were performed. Obvious LD blocks were observed in 11 of 18 QTLs (Table 3; Fig. S2). The 3B.1 QTL consisted of two blocks (3B.1.1 and 3B.1.2). The sizes of the blocks ranged from 0.5 to 23.5 cM, with an average size of 7.5 cM. By referencing Kitahonami's pedigree tree (Fig. S1), we investigated the origin and routes of transfer of QTLs into Kitahonami, based on similarities of genotypes in the LD blocks. Results indicated that the QTLs on 2B.1, 2B.2, 3D, 5D.1, 6B, 7B.1, 7B.2 and 7D were derived from the maternal variety, Kitamoe (Acc. no. 2) and 1B.2, 3B.1.1, 3B.1.2, 3B.2, 3B.3 and 4B from the paternal line, Kitakei1660 (Acc. no. 3) (Fig. 6). Since QTLs on 5A, 5D.2, 6A.1, 6A.2 and 7A existed in both parents, it could not be determined which side was the source of these QTLs in Kitahonami. QTLs originating from the maternal donor were further traced back to either Hokushin (Acc. no. 4) (3D, 7B.1, 7B.2 and 7D) or Kitakei1354 (Acc. no. 5) (2B.1, 2B.2 and 5D.1) (Fig. 6). When we attempted to trace the QTLs further back in Kitahonami's lineage, some showed discrepancies with the pedigree record (Fig. S1). However, the origins of several QTLs could be attributed to varieties introduced from abroad: it was concluded that 2B.1, 2B.2, 4B and 7D were from Ibis (Acc. no. 19), 3B.1.1 and 3B.1.2 were from Wichita (Acc. no. 12), and 3D and 5D.1 from Newthach (Acc. no. 24). The QTL on 6B seems to have originated from Norman (Acc. no. 9). The full matrix of similarities within LD blocks is shown in Table S8 in File S1.

**Table 3:** Linkage disequilibrium blocks around flour yield (FlYd) QTLs detected in this study.

		LD	Linkage	No. of	LD range (cM)		
QTL	Map^a^	block^b^	group	markers	Start	End	Size
1B.2	DArT	−	1B	2			
2B.1	SNP	−	2B	1			
2B.2	SNP	+	2B	15	202.0	217.2	15.2
3B.1.1	SNP	+	3B	16	61.0	66.4	5.4
3B.1.2	SNP	+	3B	14	68.4	71.7	3.3
3B.2	SNP	+	3B	14	89.4	94.2	4.8
3B.3	DArT	−	3B	3			
3D	DArT	−	3D	4			
4B	DArT	+	4B	10	15.0	20.4	5.4
5A	SNP	−	5A	2			
5D.1	SNP	+	5D1cult	5	42.5	47.6	5.1
5D.2	SNP	+	5D3cult	3	8.4	10.6	2.2
6A.1	SNP	+	6A	20	75.5	99.0	23.5
6A.2	SNP	+	6A	12	114.5	126.4	11.9
6B	SNP	−	6B	2			
7A	SNP	+	7A	4	58.6	63.9	5.3
7B.1	SNP	−	7B	2			
7B.2	SNP	−	7B	2			
7D	DArT	+	7D	35	1.6	2.1	0.5

a Map information is based on SNP (Cavanagh et al. [13]) and DArT (Huang et al. [23]) markers.

b Presence (+) and absence (−) of LD block.

### Validation of the QTLs with newly developed PCR markers

**Figure 7:**
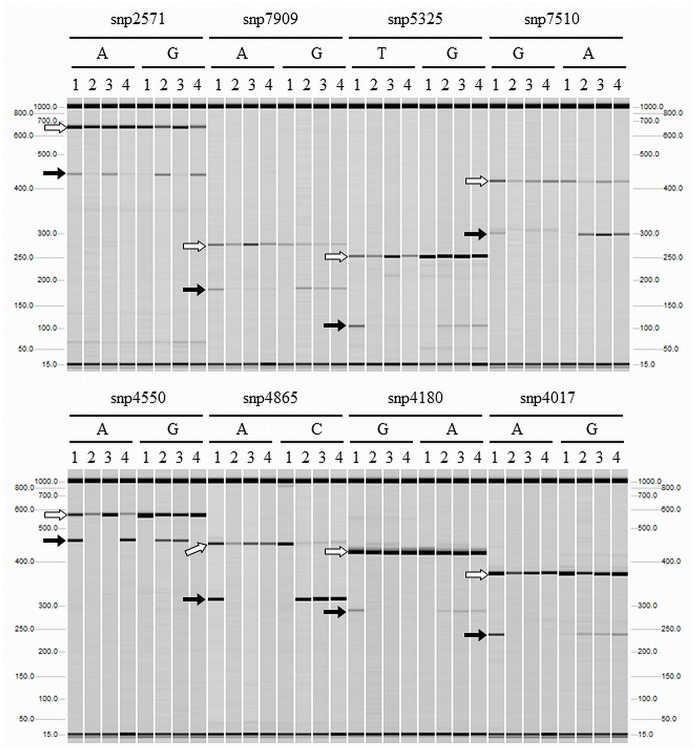
PCR assays to detect of polymorphic SNPs between Kitahonami and the three other varieties used as parents in the DH populations. White and black arrows indicate bands derived from genome-specific and allele-specific amplicons, respectively. 1: Kitahonami, 2: Kinuhime, 3: Tohoku224, 4: Shunyou.

For validation of the QTLs detected by association mapping, we performed linkage analysis using three sets of DH populations (Dataset S5 in File S2). The distribution of FlYd among lines in the three DH populations is shown in Fig. S3. For QTL validation, we wished to convert the SNP markers associated with the 11 QTLs into PCR-based markers to reduce the analysis cost involved with using the 9,000 SNP chip detection system. We succeeded in designing primer sets for eight of the 11 QTLs (Table S4 in File S1). Before use of these markers for QTL validation, their genome and allele specificity were confirmed in all four parents used for the DH populations. Based on the design of the primers, two bands were expected if the sample sequence matched with the sequence of the ASP, and a single band if it did not. All amplified products showed the expected band patterns (Fig. 7), indicating the new PCR-based markers were capable of identifying the eight QTLs.

Using these markers, it was determined that four of the QTLs had significant effects on FlYd in the Kinuhime/Kitahonami population, three had significant effects in the Tohoku224/Kitahonami population, and four QTLs had significant effects in the Shunyou/Kitahonami population (Table 4). Notably, QTLs on 3B.1.1, 3B.2 and 7A showed highly significant and consistent effects across the populations.

**Table 4:** Validations of flour yield (FlYd) QTLs using three DH populations.

QTL	Marker^a^	Allele^b^	Kinuhime/Kitahonami			Tohoku224/Kitahonami			Shunyou/Kitahonami		
			Number	Mean	p	Number	Mean	p	Number	Mean	p
2B.1	snp2571	A	79	63.01	0.559	160	64.14		75	66.69	0.694
		G	72	63.31					80	66.51	
2B.2	snp7909	A	86	63.75	0.007^**^	80	64.55	0.091	76	66.82	0.323
		G	65	62.36		80	63.73		79	66.38	
3B.1.1	snp5325	T	70	63.92	0.005^**^	67	65.24	<.0001^***^	71	67.66	<.0001^***^
		G	81	62.49		93	63.35		83	65.69	
3B.2	snp7510	G	72	63.77	0.022*	68	65.05	0.001^**^	66	67.34	0.004^**^
		A	79	62.59		92	63.47		89	66.05	
5D.1	snp4550	A	81	63.23	0.755	87	64.19	0.844	155	66.60	
		G	70	63.07		73	64.09				
6A.2	snp4865	A	74	63.56	0.117	75	64.20	0.811	75	66.78	0.408
		C	77	62.76		85	64.09		77	66.40	
7A	snp4180	G	71	64.07	0.001^***^	76	64.89	0.003^**^	84	67.12	0.011*
		A	80	62.34		84	63.46		71	65.98	
7B.1	snp4017	A	82	63.43	0.246	75	64.52	0.138	84	67.29	0.001^***^
		G	69	62.83		85	63.81		70	65.77	

a Numbers in marker names correspond to the index of Illumina's 9K Infinium array (Cavanagh et al. [13]).

b Kitahonami's allele is shown at the top for each marker.

*, ** and *** indicate significant differences between allele mean values at 5%, 1% and 0.1% level, respectively.

**Figure 8:**
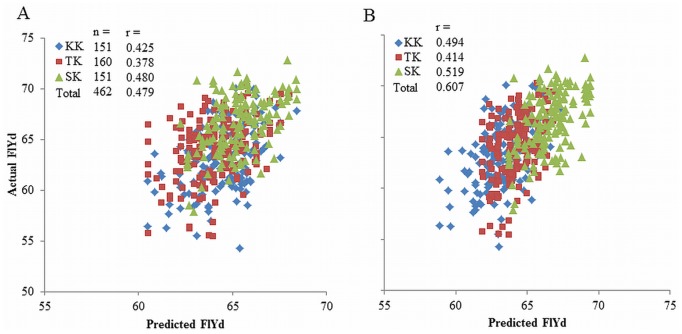
Scatter diagrams of predicted and actual FlYd values in DH lines. Regression models were constructed using the three DH populations together (DH_total) (A) or separately (DH_each) (B). KK: Kinuhime/Kitahonami, TK: Tohoku224/Kitahonami, SK: Shunyou/Kitahonami population.

Multiple regression analysis showed significant effects in five of the eight markers using the DH_total dataset, which is based on the combination of all three DH populations (Table S9 in File S1). For the DH_each dataset, based on individual DH populations, four, two and three markers were significant in Kinuhime/Kitahonami, Tohoku224/Kitahonami and Shunyou/Kitahonami populations, respectively (Table S9 in File S1). The regression models were used to predict FlYd in the individual DH lines (Dataset S5 in File S2). When we considered all DH lines together, the correlation coefficients between predicted and actual values were 0.479 for DH_total and 0.607 for DH_each (Fig. 8). In each population, prediction accuracies based on the DH_each dataset were consistently higher than those based on the DH_total dataset (Fig. 8).

## Discussion

Array-based systems allow genotyping using a large number of markers simultaneously, and the use of these systems has become popular for rapidly determining the genetic diversity and population structure of samples [13], [24]–[26]. The DArT and SNP arrays used in this study contain approximately 7,000 and 9,000 markers, respectively. Among these markers, 42% of the DArT and 67% of the SNP markers showed polymorphisms among the accessions used here. By using these high-density genome-wide markers, we could provide the first precise overview of genetic variation in Japanese wheat varieties (Fig. 1; Fig. 2). The 185 lines used in the diversity analysis included an association panel of 65 lines. As expected, the lines in the association panel showed a higher level of similarity compared to other accessions, which is reasonable given that the association panel consists mainly of lines developed at KAES in the Hokkaido region; most are winter lines that are well-adapted to the northern region of Japan. Such regional adaptation is an important feature in evaluating genetic performances of complex traits such as yield and grain quality. Of the 33 lines in the pedigree record of Kitahonami (Fig. S1), 24 accessions were still available and were employed in this study. The cladograms generated by SNP markers clearly showed that Kitamoe (Acc. no. 2), Kitakei1660 (3), Hokushin (4) and Kitakei1354 (5) were clustered close to Kitahonami (Fig. 2), agreeing with the pedigree record. This indicates that kinship matrix generated with markers can be useful for representing the familial relation of accessions.

The analysis of variance indicated there was significant genetic and environmental variation for the target trait (Fig. 3). Samples collected at Morioka in 2010 showed the lowest mean FlYd values compared to other environments. The meteorological data recorded during the cropping seasons did not indicate any clear reason for this (data not shown). Although the differences among mean values between environments were significant (Fig. 3), the relative differences among accessions were consistent over the environments (Table 1). For example, Kitahonami consistently grouped within the eight accessions showing highest values for FlYd in all environments, indicating that Kitahonami carries alleles affecting this trait that will be useful across environments.

In the diversity analysis, the lines used for association mapping did not fall into distinct groups, but did show high familial relatedness. Therefore, we performed association mapping using kinship matrix (K) rather than population structure (Q) as a covariant. Based on a plot of expected versus observed p values, this correction achieved a reduction in the false positive rate (data not shown). Kernel type is known to have a great impact on milling yield traits [3], and the *Pin* genes, which encode puroindolines, determine kernel type [27], [28]. In this study, a significant difference in mean values of FlYd was observed between soft and hard accessions grouped by *Pina-D1* and *Pinb-D1* genotypes (Fig. 4). Therefore, kernel type was used as a fixed effect term in the statistical model. This treatment resulted in a major improvement in the association mapping results. When kernel type was not considered, no significant MTAs were detected at a q value of 0.5. However, when kernel type was used as a covariant, 62 MTAs were identified at this q value. This implies that the statistical model employed in this study, which used both kinship and kernel type as covariants, was appropriate for identifying genetic factors related to FlYd in Kitahonami.

It was not possible to precisely compare the positions of QTLs detected in this study to those identified in previous reports, since few identical markers were used. However, based on the microsatellite consensus map [29], the QTLs on 2B.1, 2B.2, 3B.2, 6A.2 and 7A observed here may correspond to those reported by Smith et al. [4], Lehmensiek et al. [5], Carter et al. [9], Fox et al. [6] and Lehmensiek et al. [5], respectively. The pedigree analysis showed that ongoing pyramiding of QTLs had occurred during the history of wheat breeding in the Hokkaido region. The high FlYd values of Kitahonami were achieved by combining eight positive QTLs from maternal lines, six from paternal lines, and five from both sides (Fig. 6). This information will be useful in developing effective breeding strategies to improve FlYd, since it allows us to predict the performance of progeny lines from a specific cross based on the genotypes of parental varieties or lines.

The level of LD can be affected by various factors including linkage, selection, and admixture [30]. Although there have been several studies on LD levels in various wheat populations [7], [31]–[35], direct comparisons between studies is difficult, since LD levels are influenced by the type of markers used for genotyping and by sample size. However, generally LD decays to half of the initial value within less than 9 cM. In this study, LD blocks with more than 10 cM were identified in the QTLs on 2B.2, 3B.1+3B.2 (these two QTLs are closely linked), 6A.1 and 6A.2. Since the accessions in this study consist mainly of breeding materials, it is possible that LD blocks detected in the QTL regions result from selection for favorable phenotypes during the history of wheat breeding in KAES.

Segregation analysis confirmed that five of the eight QTLs tested had significant effects on the FlYd (Table 4). Previous studies using bi-parental populations reported that flour yield QTLs were detected on most wheat chromosomes but it was not demonstrated whether these QTLs maintained their favorable effects in materials with different genetic backgrounds. In this study, we used three different populations in which Kitahonami served as pollen donor to confirm positive effects for five out of eight QTLs. The contributions of 3B.1.1, 3B.2 and 7A were significant in all three populations, and the contributions of 2B.2 and 7B.1 were significant in one population. Although the effects of 2B.1, 5D.1 and 6A.2 were not confirmed in the three DH populations, this does not mean these QTLs have no positive effects on FlYd; rather, they have significant effects in a specific genetic background. Generally, it can be expected that during long term breeding programs, positive QTLs will accumulate in most breeding materials. However, the usefulness of these QTLs for crop improvement via breeding will be determined by their robustness, or their ability to predict effects in a range of genetic backgrounds. In this study, the QTLs on 3B and 7A consistently showed highly significant effects across three DH populations (Table 4). QTL analyses using a joint linkage map from these three populations indicated that the 3B and 7A QTLs explained 6.0% and 11.7% of the total variation, respectively (data not shown). Besides being significant in the DH populations, these QTLs were also present in lines with high flour yield originating from three separate breeding programs where Kitahonami was used as a parent (data not shown).

The effects of the markers on FlYd were not identical between single and multiple regression models (Table 4; Table S9 in File S1). These differences may be caused by relationships among the eight markers, since there were significant relationship between snp2571 and snp7909, between snp2571 and snp4550, and between snp5325 and snp7510 (data not shown). The correlation coefficients between actual and predicted values indicated that the model based on the DH_each dataset showed higher prediction accuracy than that based on the DH_total (Fig. 8). This result implies that the model should be constructed based on each cross combination. This is reasonable, because the number and effects of QTLs segregating in a biparental population varied within crosses. Since the three DH populations in this study share Kitahonami as a paternal parent, the differences observed between the two regression models among populations were caused by the differences in genetic backgrounds of maternal parents. In this study, the multiple regression analysis indicated that a relatively high prediction accuracy (r = 0.607) was achieved when the eight markers were applied for each DH population at the same time (Fig. 8). Not all markers showed positive effects in each DH population, yet the prediction accuracy was higher when all markers were used concurrently, as opposed to using only those markers that showed a positive effect for a specific DH population. Therefore, in terms of practical breeding, the construction of a regression model using all QTLs identified by GWAS in this study represents an attractive approach for increasing the selection efficiency for FlYd. Notably, the prediction accuracy was higher when the multiple regression model was based on data from each DH population (r = 0.607), rather than on data from combining the three DH populations (0.479) or data from the panel (0.370).

In this study, PCR-based markers linked to eight QTLs were developed (Fig. 7; Table S4 in File S1). Recently, large numbers of SNPs have been identified and characterized in wheat [13], [24], [36]–[38]. Using these resources, high density array-based markers have been established and used for diversity and LD analyses. Those tools have opened a new gate for understanding the genetic architecture of populations of interest. However, although the cost of genotyping per data point has dramatically declined, array-based systems are still relatively costly to access. This hinders the adoption of array-based systems in crop breeding programs, especially in the public sector. Since PCR-based markers are commonly used in MAS and are well suited to breeding programs, we decided to convert array-based SNP markers to PCR-based markers. In hexaploid wheat, difficulties arise in distinguishing allelic from genomic SNPs [38]. This is especially problematic because most SNP resources originate from exonic sequences [39], [40] which tend to maintain higher similarity among A, B and D genomes than intronic sequences. This led us to design not only allele specific but also genome specific primers for precise targeting of SNPs. The chromosome locations of the target SNPs and the corresponding probe sequences were used to sort out the A, B and D homoeologous contigs from chromosome-arm specific survey sequences (IWGSC, http://www.wheatgenome.org/) [18]. By aligning the three contig sequences, we could identify polymorphic positions flanking the target SNP. These regions were used to design genome specific primer sets, allowing the amplification of fragments specific to the genome from which the SNP originated. Only eight markers were developed in this study, but using the same strategy we have succeeded in developing PCR-based markers capable of detecting 54 additional SNPs. We estimate that approximately 70% of the publicly available wheat SNP markers can be converted to genome specific PCR-based markers.

Milling tests require a substantial quantity of grain, meaning that selection for flour yield cannot be performed at the early stages of wheat breeding programs. The identification of markers linked to the flour yield trait can circumvent this problem. Using association mapping with a mixed model, we identified 21 QTLs influencing flour yield. The role of these QTLs was supported by pedigree information and results of linkage analysis. Notably, we identified several QTLs which were consistently associated with high flour yield across different genetic backgrounds. The introduction of these QTLs from Kitahonami into other lines by MAS is a promising method of improving flour yield in Japanese soft wheat varieties.

## Supporting Information

Figure S1 **Record of Kitahonami's pedigree.**
(PDF)

Figure S2 **LD charts produced by TASSEL 3.0.**
(PDF)

Figure S3 **Distribution of FlYd in the three DH populations.**
(PDF)

File S1 **Supporting tables.** Table S1, List of accessions used in this study. Table S2, Distribution of the markers used for association mapping across the wheat genome. Table S3, Description of traits investigated. Table S4, Details of PCR-based markers developed in this study. Table S5, Variance components and heritabilities of traits investigated. Table S6, Correlation coefficients among traits. Table S7, List of marker-trait associations (MTAs) detected by genome-wide association mapping. Table S8, Full matrix produced by similarity analysis in LD blocks. Table S9, Results of multiple regression analysis using the three DH populations.
(XLSX)

File S2 **Datasets.** Dataset S1, DArT genotyping data for diversity analyses. Dataset S2, SNP genotyping data for diversity analyses. Dataset S3, Phenotyping data for association mapping. Dataset S4, Genotyping data for association mapping. Dataset S5, Genotype and phenotype data of doubled haploid populations.
(ZIP)
