# Supplementary figures and images for: Association Mapping and Validation of QTLs for Flour Yield in the Soft Winter Wheat Variety Kitahonami
Source: PLoS One. 2014 Oct 31;9(10):e111337. doi: 10.1371/journal.pone.0111337 (PMC4215981; doi:10.1371/journal.pone.0111337)

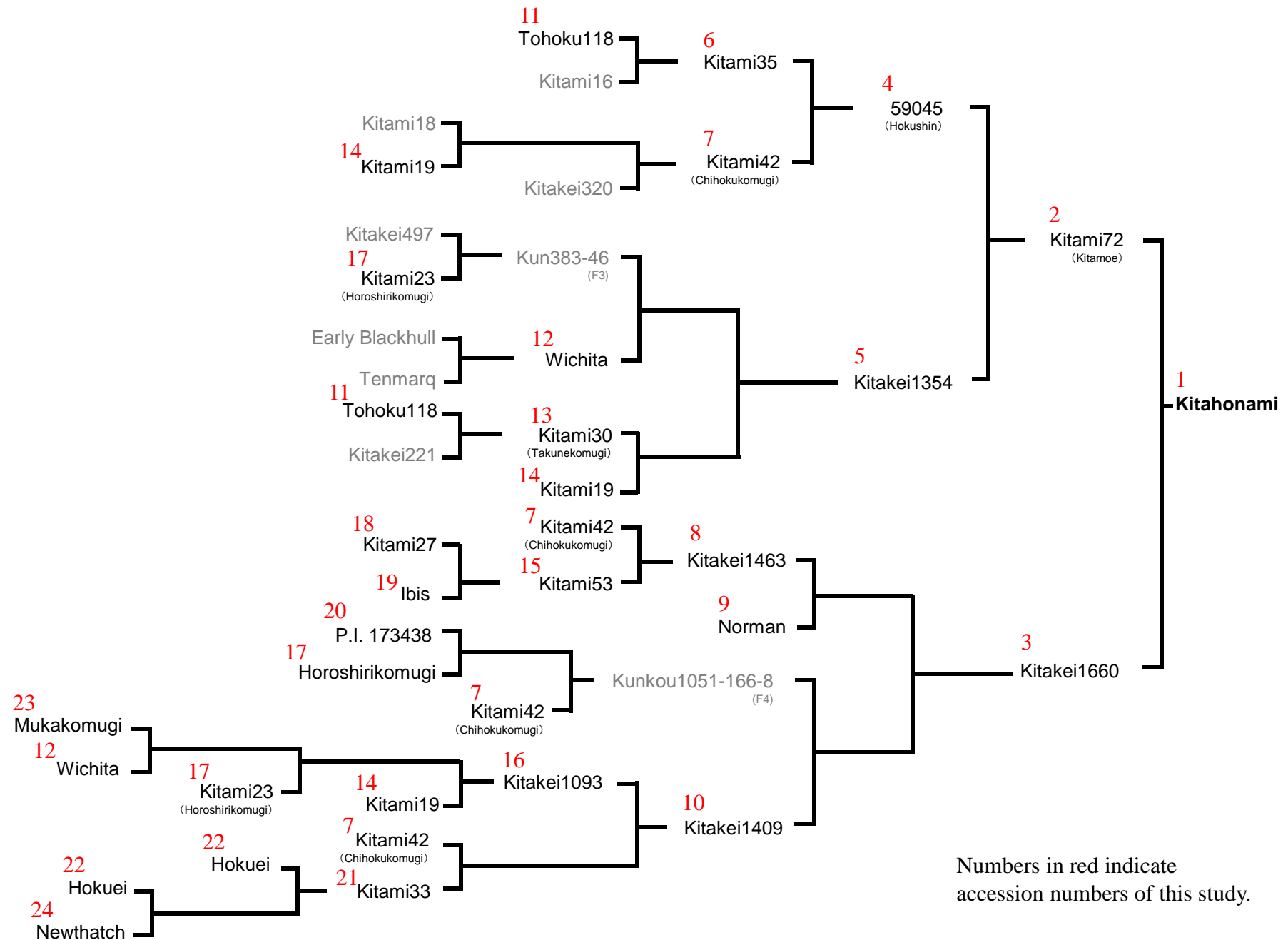

Supplement: Figure S1 — Record of Kitahonami's pedigree. (PDF) [file pone.0111337.s001.pdf]

Orange circles indicate positions of significant markers for flour yield.

2B.2

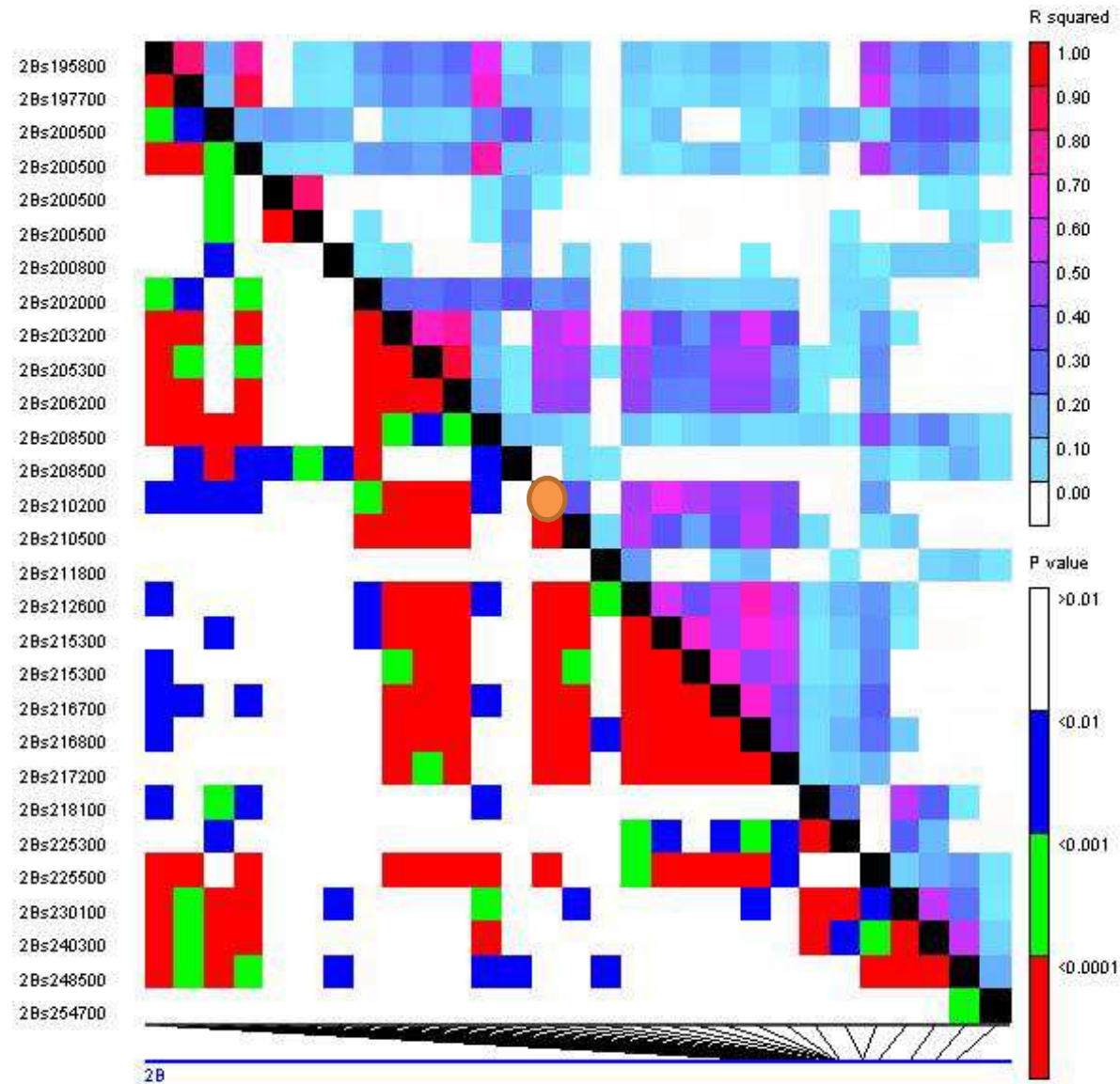

### 3B.1.1

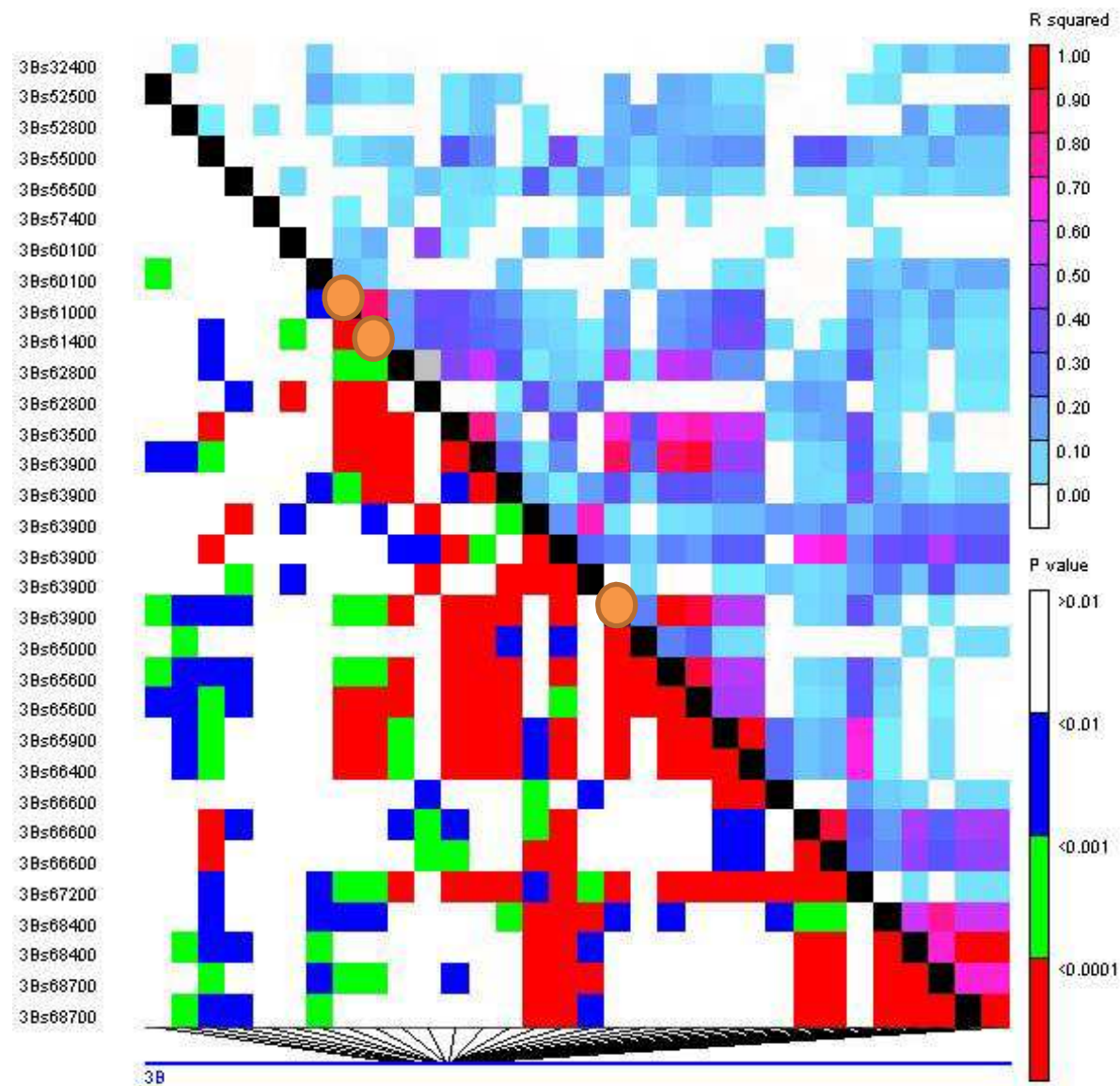

### 3B.1.2

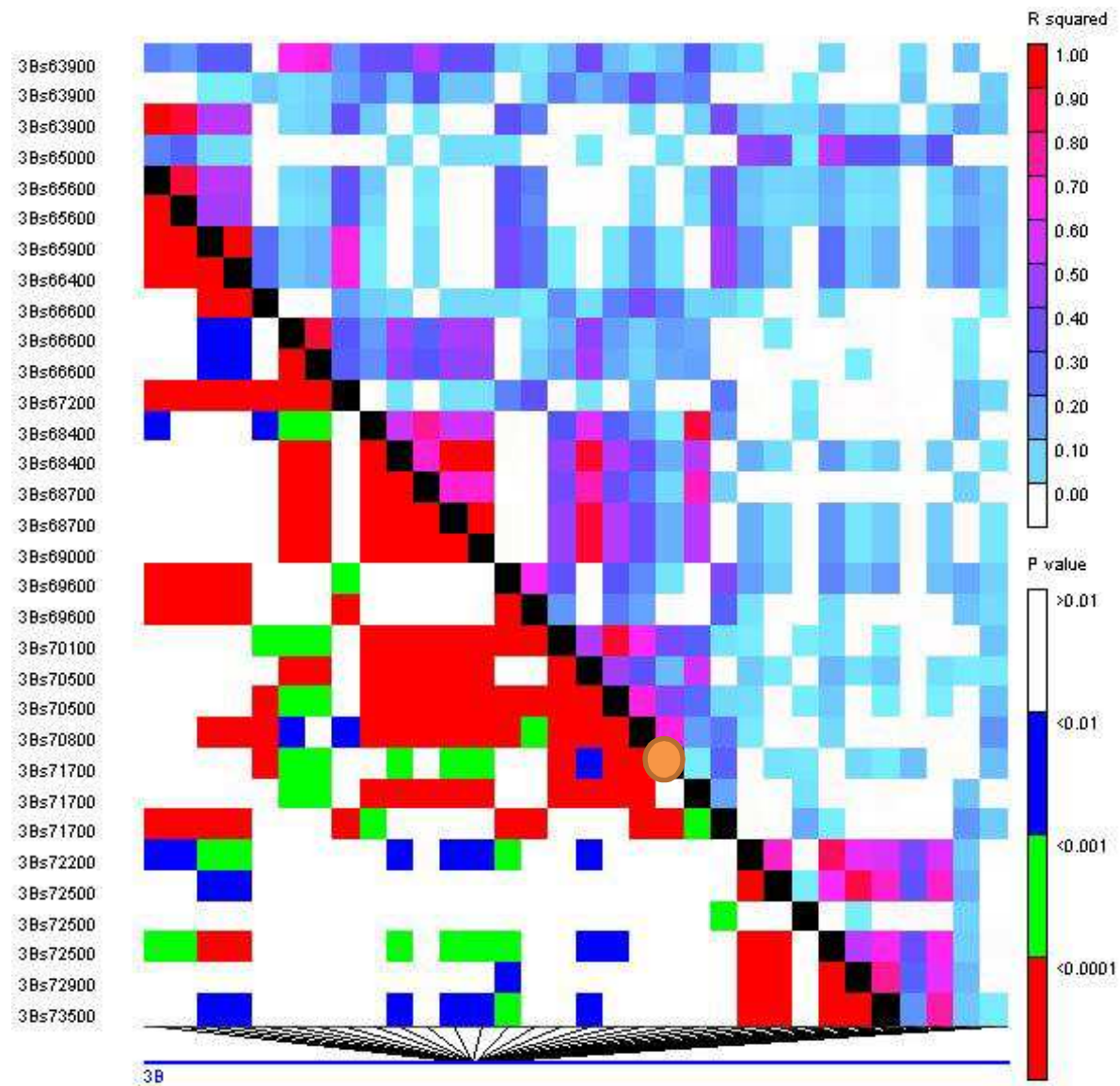

3B.2

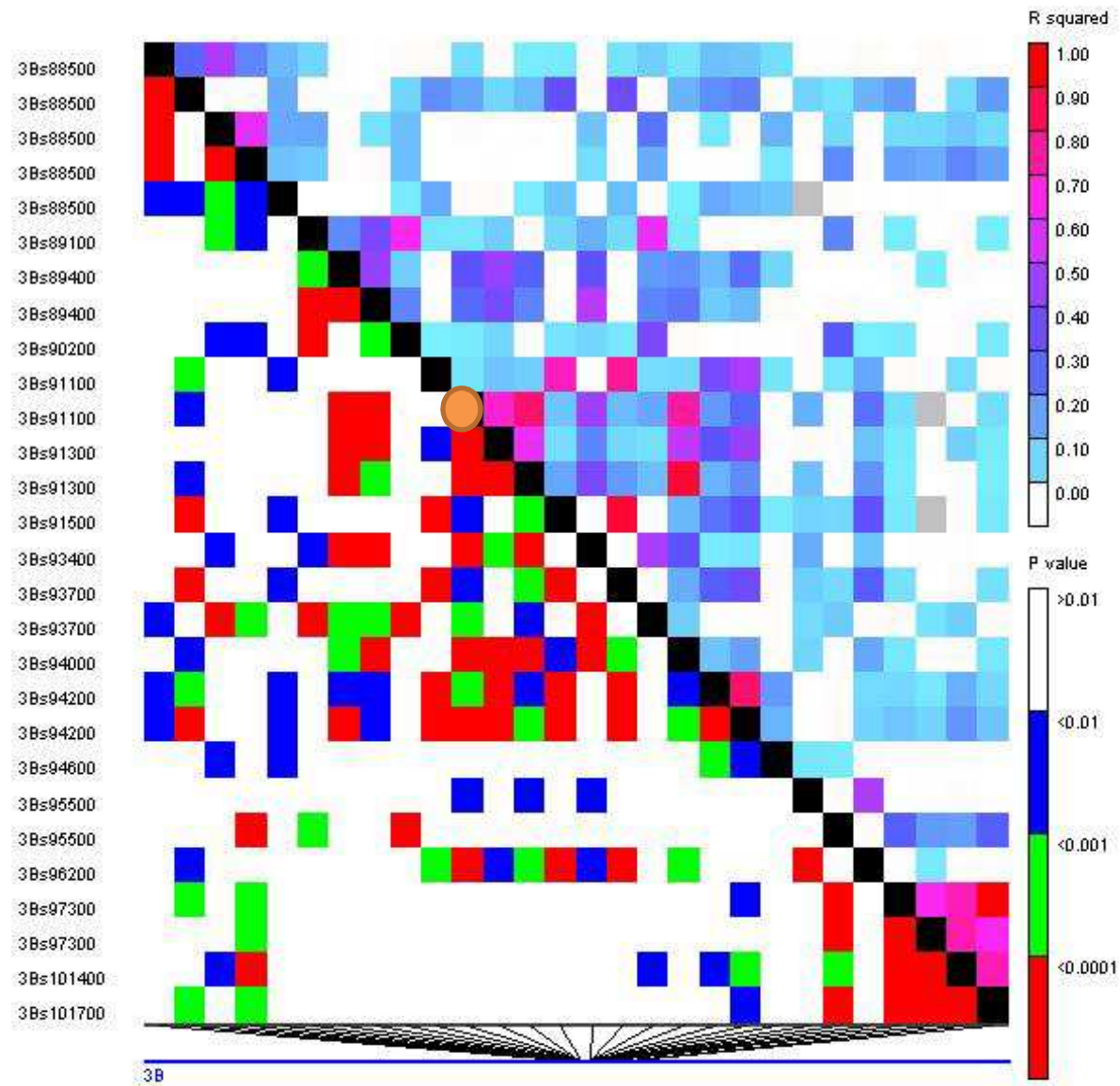

4B

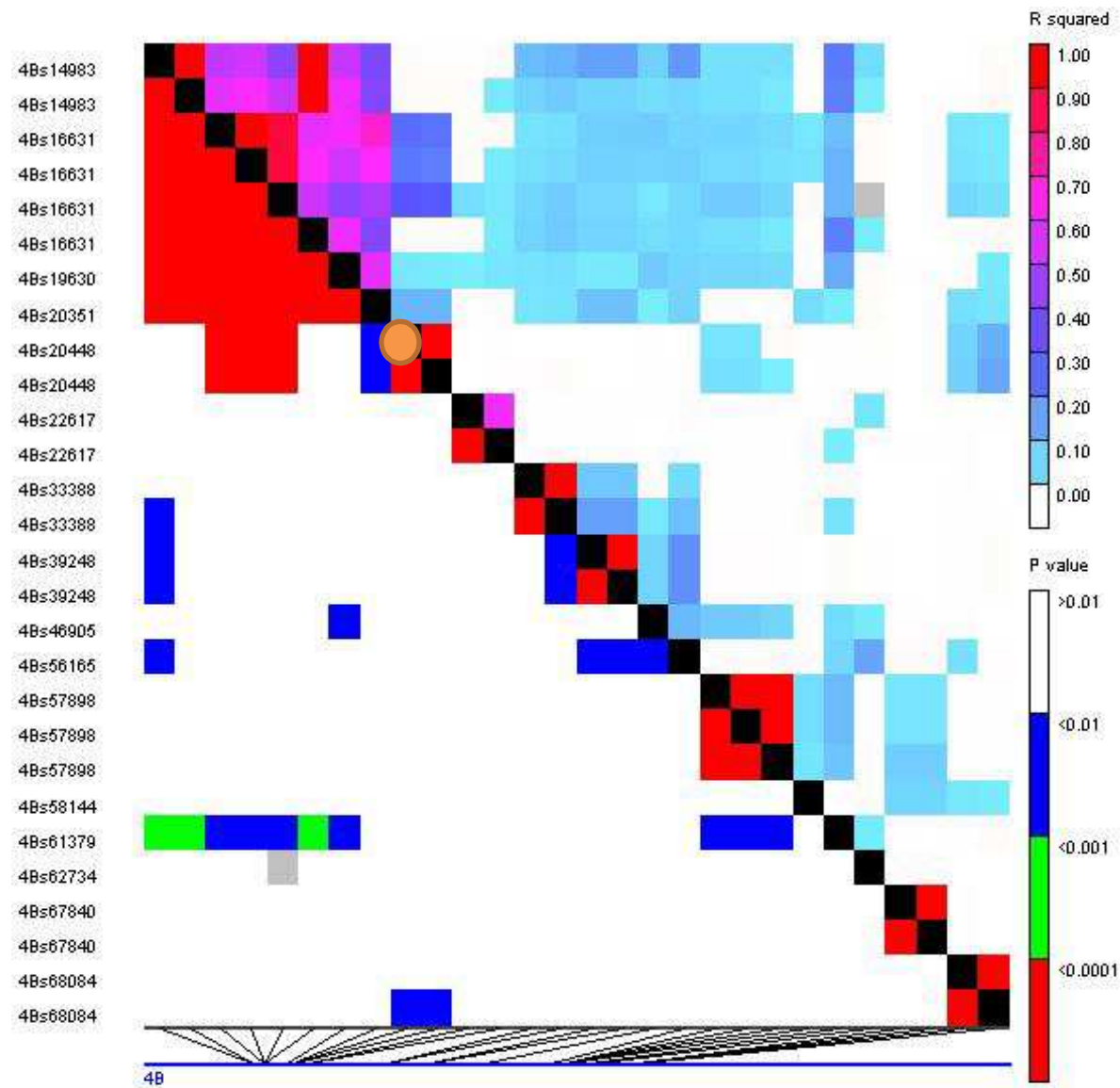

## 5D.1

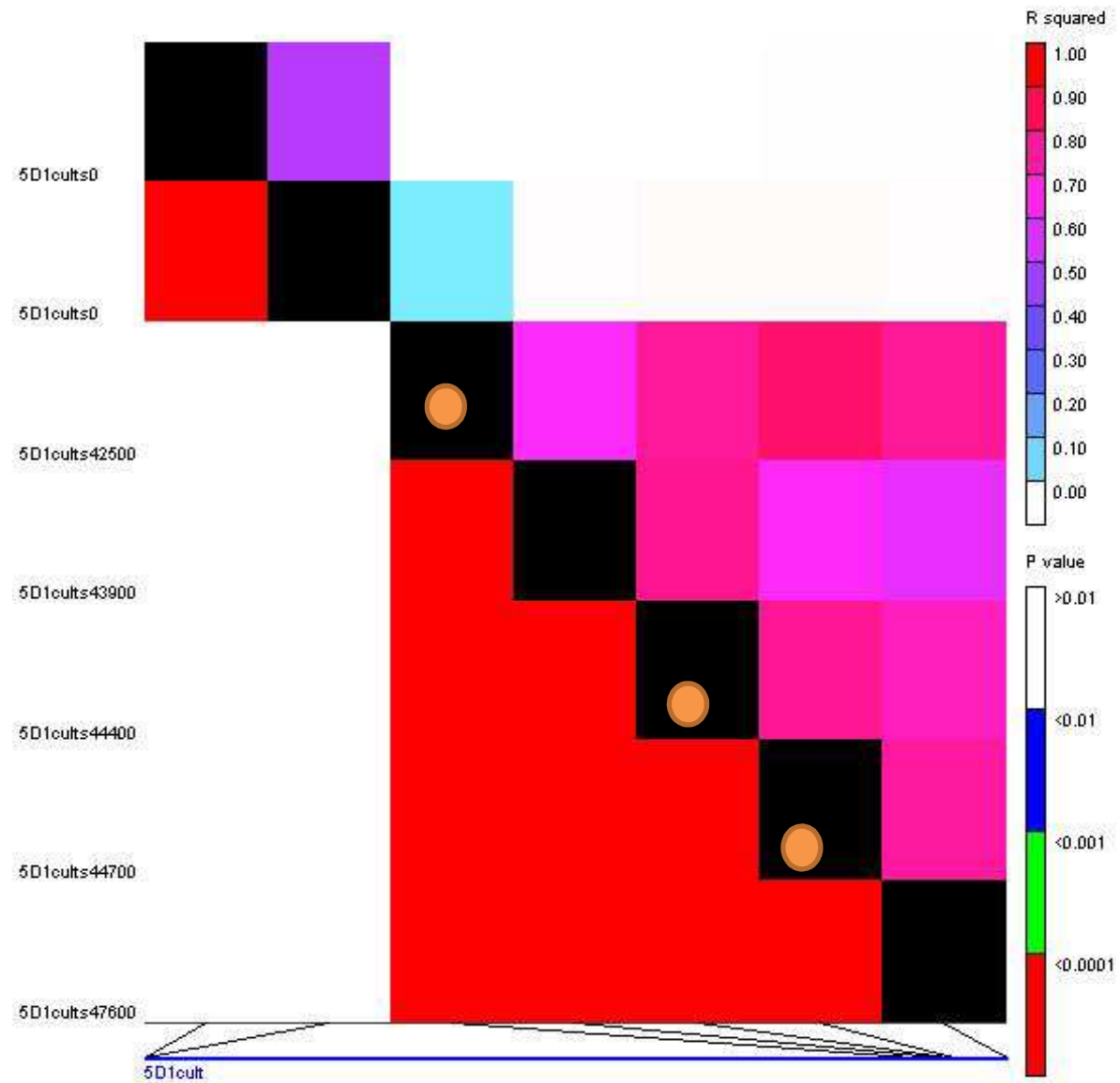

5D.2

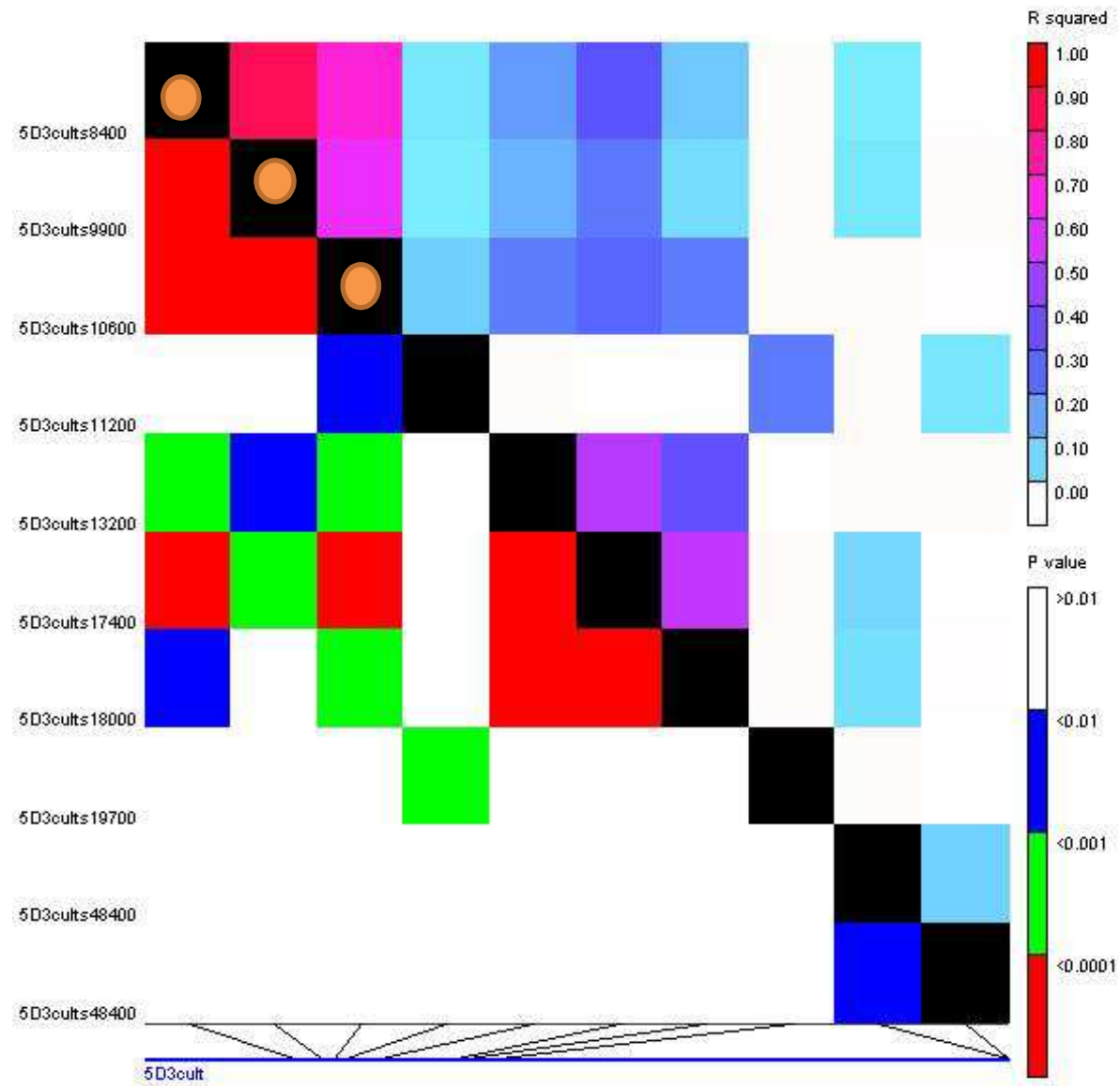

6A.1

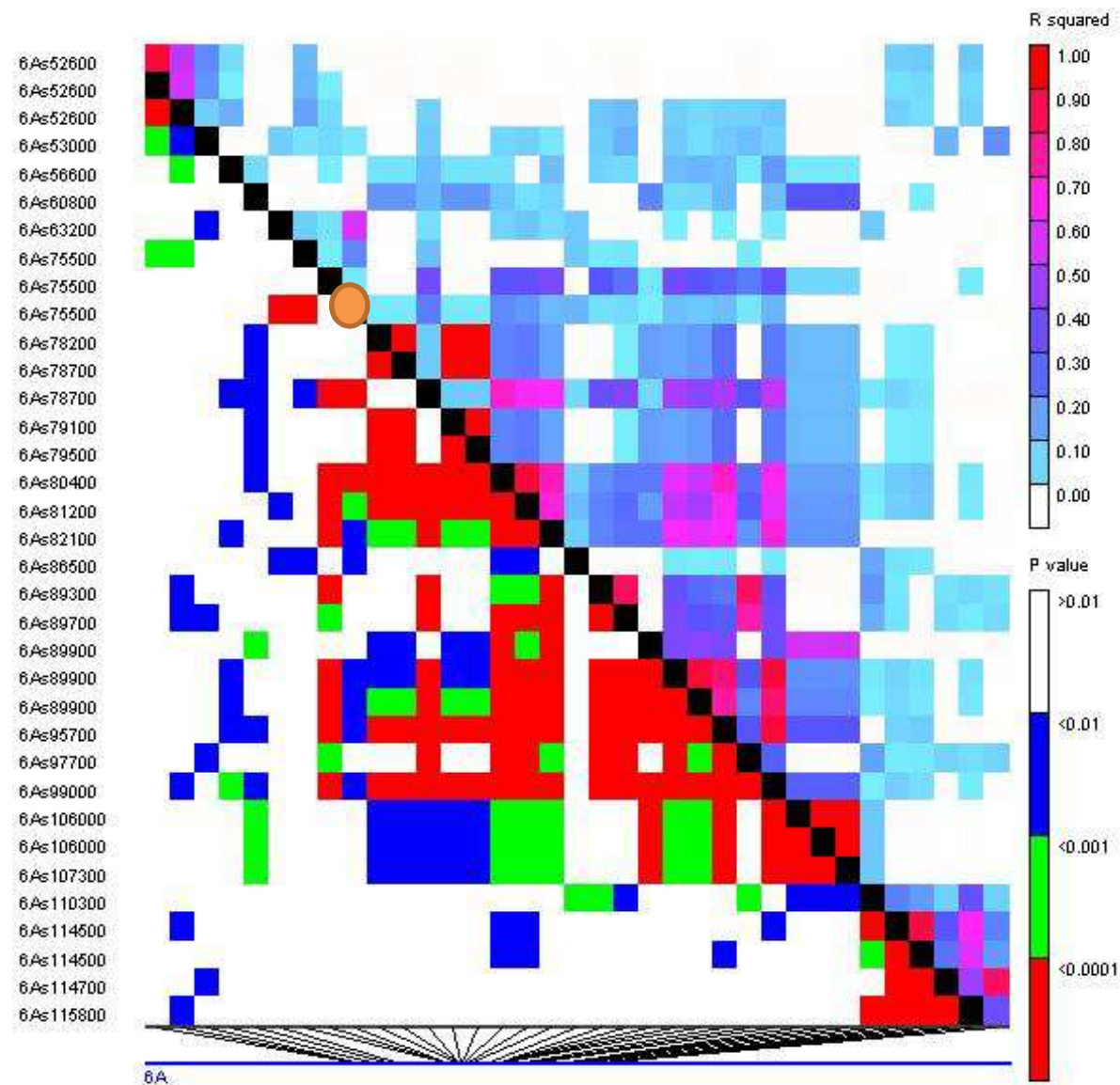

6A.2

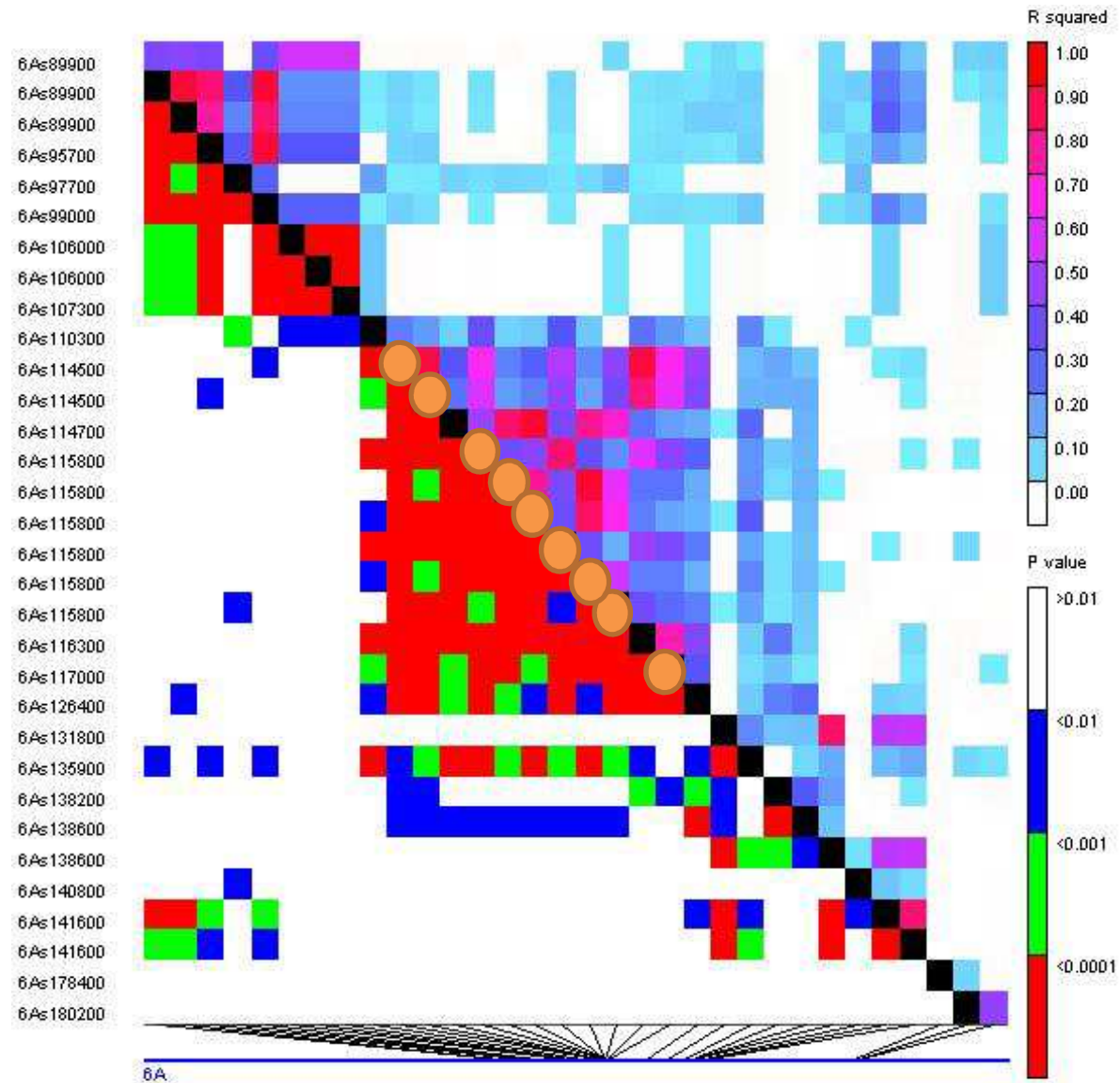

7A

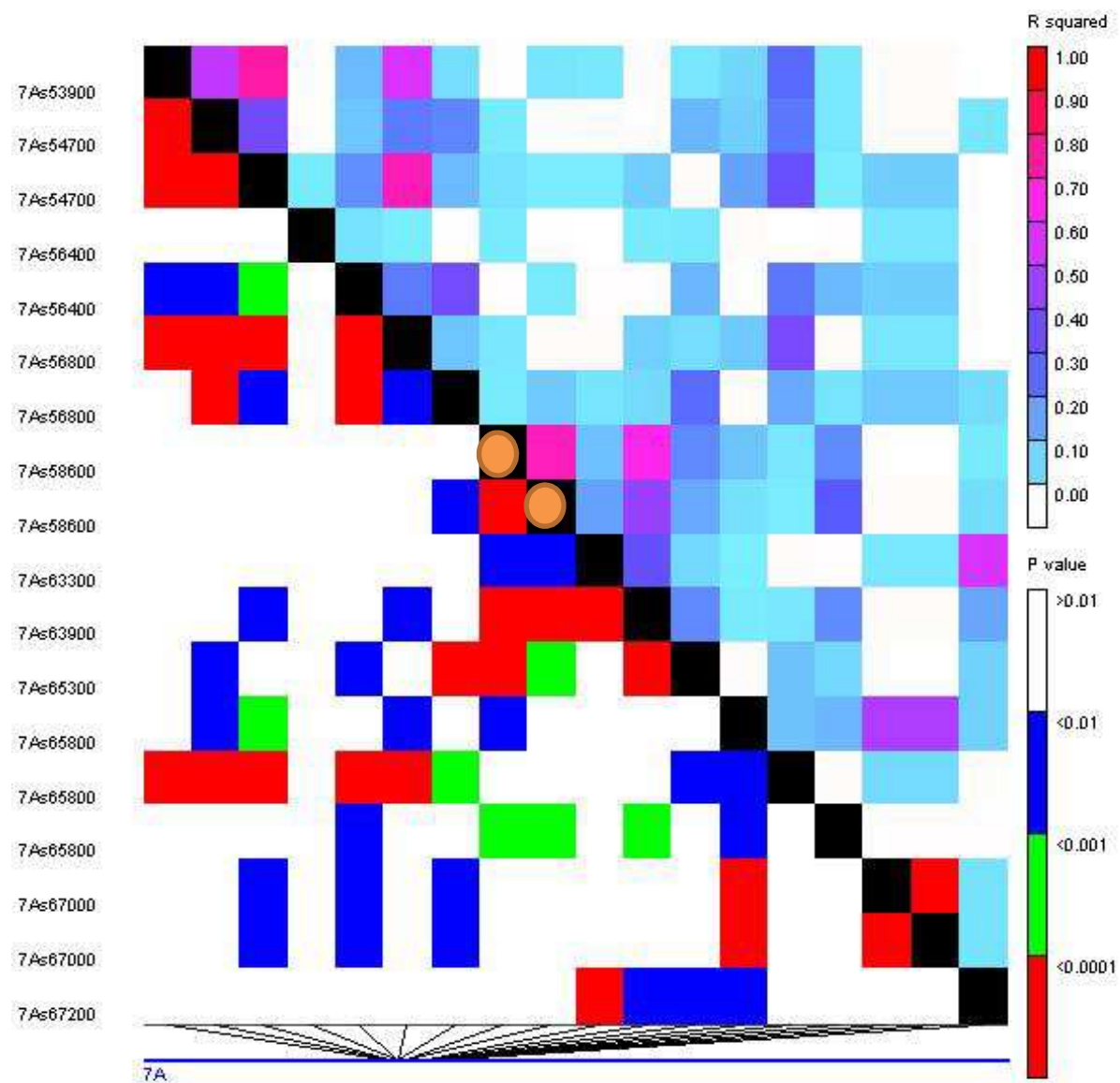

7D

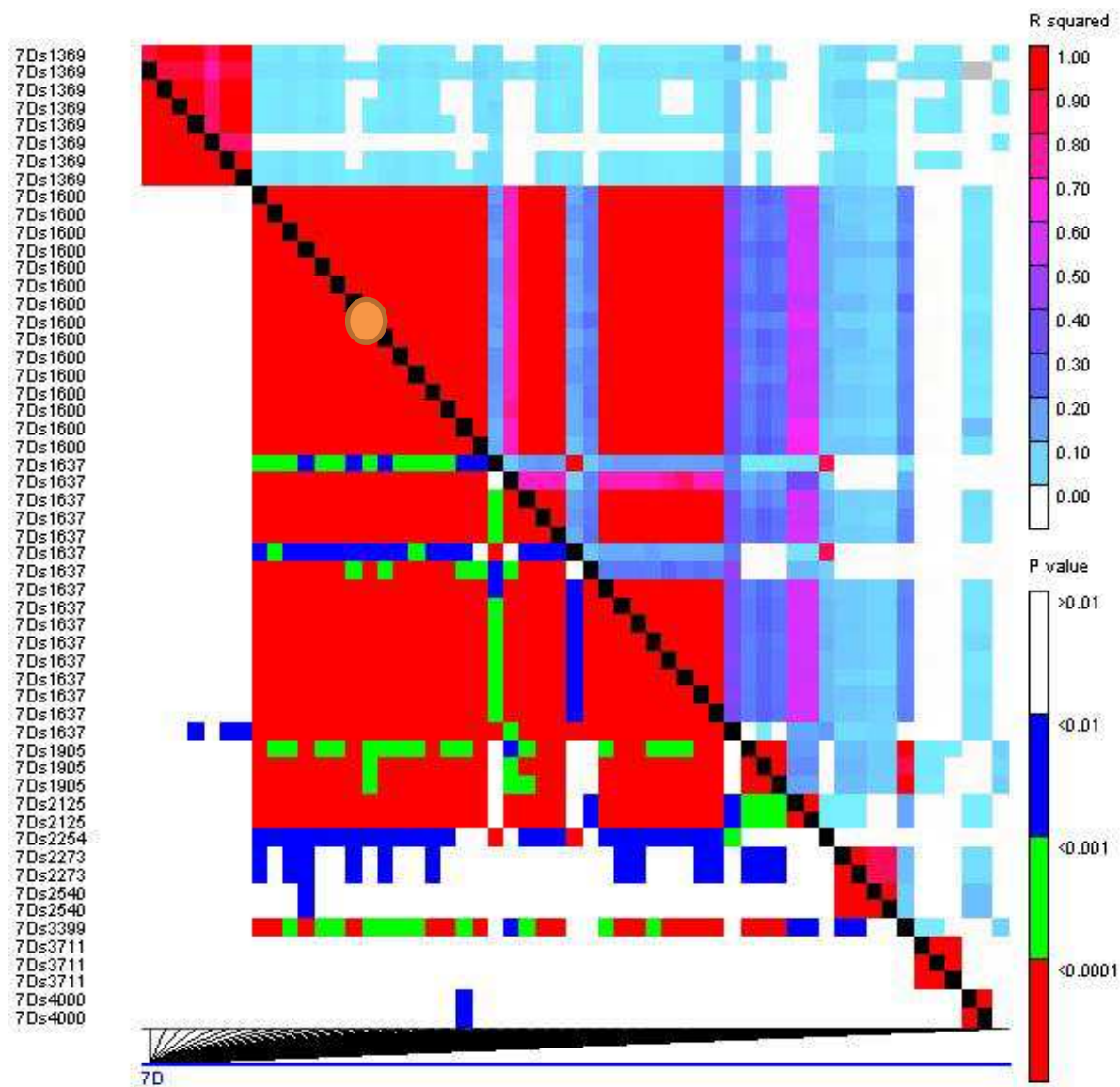

Supplement: Figure S2 — LD charts produced by TASSEL 3.0. (PDF) [file pone.0111337.s002.pdf]

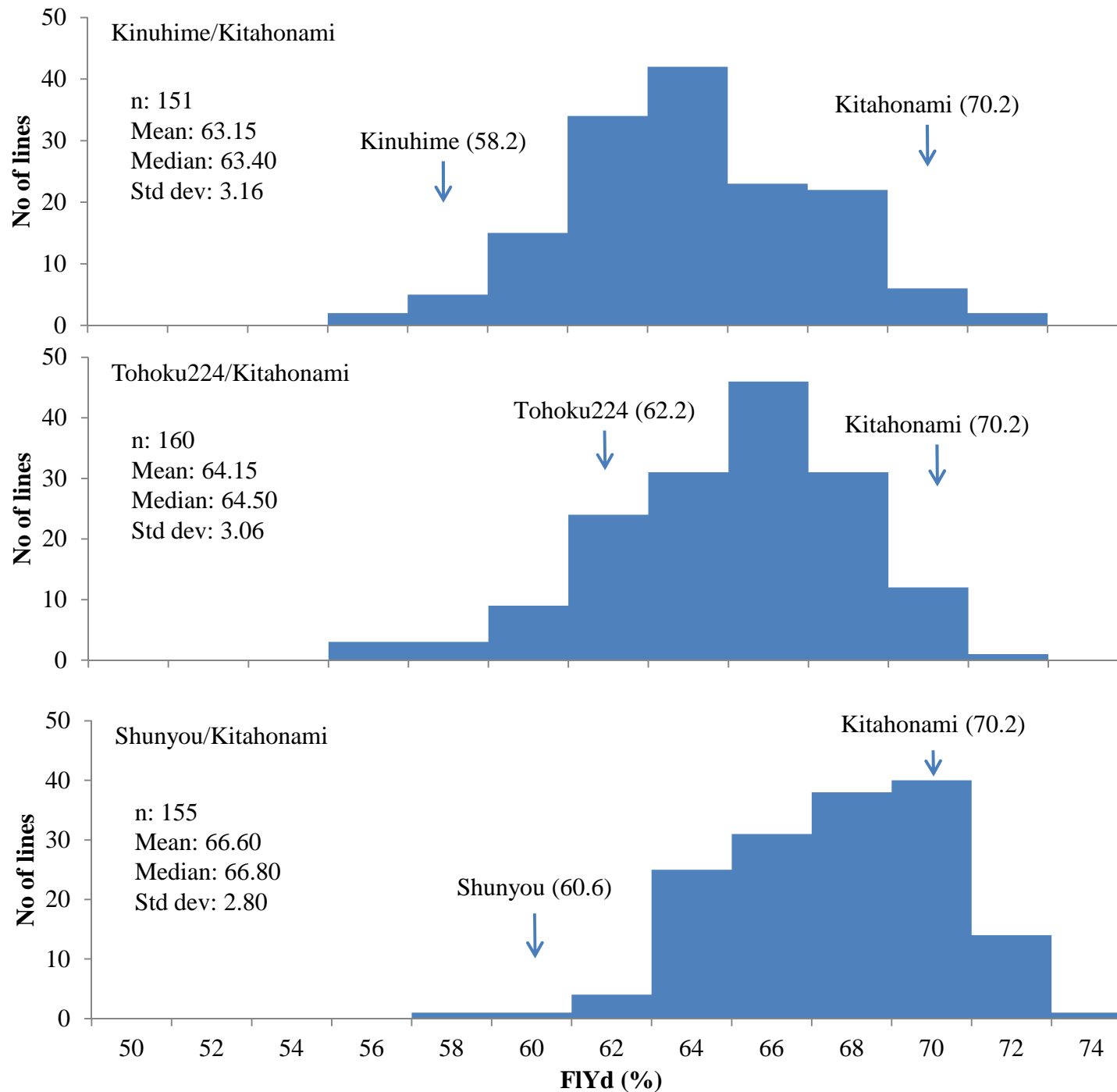

Supplement: Figure S3 — Distribution of FlYd in the three DH populations. (PDF) [file pone.0111337.s003.pdf]
